# Whole exome sequencing in 75 high-risk families with validation and replication in independent case-control studies identifies *TANGO2*, *OR5H14*, and *CHAD* as new prostate cancer susceptibility genes

**DOI:** 10.18632/oncotarget.13646

**Published:** 2016-11-26

**Authors:** Danielle M. Karyadi, Milan S. Geybels, Eric Karlins, Brennan Decker, Laura McIntosh, Amy Hutchinson, Suzanne Kolb, Shannon K. McDonnell, Belynda Hicks, Sumit Middha, Liesel M. FitzGerald, Melissa S. DeRycke, Meredith Yeager, Daniel J. Schaid, Stephen J. Chanock, Stephen N. Thibodeau, Sonja I. Berndt, Janet L. Stanford, Elaine A. Ostrander

**Affiliations:** ^1^ National Human Genome Research Institute, National Institutes of Health, Bethesda, MD, USA; ^2^ Division of Public Health Sciences, Fred Hutchinson Cancer Research Center, Seattle, WA, USA; ^3^ Division of Cancer Epidemiology and Genetics, National Cancer Institute, National Institutes of Health, Bethesda, MD, USA; ^4^ Department of Health Sciences Research, Mayo Clinic, Rochester, MN, USA; ^5^ Menzies Institute for Medical Research, University of Tasmania, Hobart, Australia; ^6^ Department of Laboratory Medicine and Pathology, Mayo Clinic, Rochester, MN, USA; ^7^ Department of Epidemiology, School of Public Health, University of Washington, Seattle, WA, USA

**Keywords:** whole exome sequencing, cancer susceptibility, high-risk families, case-control association, prostate cancer

## Abstract

Prostate cancer (PCa) susceptibility is defined by a continuum from rare, high-penetrance to common, low-penetrance alleles. Research to date has concentrated on identification of variants at the ends of that continuum. Taking an alternate approach, we focused on the important but elusive class of low-frequency, moderately penetrant variants by performing disease model-based variant filtering of whole exome sequence data from 75 hereditary PCa families. Analysis of 341 candidate risk variants identified nine variants significantly associated with increased PCa risk in a population-based, case-control study of 2,495 men. In an independent nested case-control study of 7,121 men, there was risk association evidence for *TANGO2* p.Ser17Ter and the established *HOXB13* p.Gly84Glu variant. Meta-analysis combining the case-control studies identified two additional variants suggestively associated with risk, *OR5H14* p.Met59Val and *CHAD* p.Ala342Asp. The *TANGO2* and *HOXB13* variants co-occurred in cases more often than expected by chance and never in controls. Finally, *TANGO2* p.Ser17Ter was associated with aggressive disease in both case-control studies separately. Our analyses identified three new PCa susceptibility alleles in the *TANGO2*, *OR5H14* and *CHAD* genes that not only segregate in multiple high-risk families but are also of importance in altering disease risk for men from the general population. This is the first successful study to utilize sequencing in high-risk families for the express purpose of identifying low-frequency, moderately penetrant PCa risk mutations.

## INTRODUCTION

Prostate cancer (PCa) is the most common non-cutaneous tumor in men from the United States with 180,890 estimated new cases and 26,120 expected deaths in 2016 [1]. Epidemiological studies of twins suggest that PCa has a strong genetic component with approximately 42% to 58% of risk attributed to genetic factors [2, 3]. The disease is genetically heterogeneous and predicted to be caused by a continuum from common, low-penetrance to rare, high-penetrance variants [4]. Genome-wide association studies (GWAS) have identified over 100 loci associated with PCa risk in men of European ancestry [5]. When combined these loci are predicted to account for approximately 33% of PCa familial risk [5]. Genes with moderately penetrant variants have also been associated with increased disease risk, but to date, only *BRCA2* and *HOXB13* have been consistently implicated [4, 6–10]. Given the rarity of the *BRCA2* and *HOXB13* variants in the general population, these loci likely account for only a small proportion of the PCa genetic risk [4, 8]. As such, a significant proportion of the heritability of PCa risk remains undiscovered.

Whole exome sequence (WES) of hereditary PCa (HPC) families represents a unique resource to identify low-frequency, moderately penetrant variants which, when considered in aggregate, could contribute substantially to PCa susceptibility. In this study, we performed an analysis of WES data from 75 HPC families. We designed the analysis strategy to identify putative risk variants, taking into account the likely genetic heterogeneity and incomplete penetrance of PCa susceptibility alleles. We first assumed that, due to genetic heterogeneity, only a few families would share any particular candidate variant and that any candidate variant was unlikely to be present in every affected man in a carrier family. We therefore utilized data regarding the frequency of risk allele carriers in all affected men. Second, we assumed that PCa causal variants segregating in families would most likely be moderately rather than highly penetrant and, as such, some of the cases' unaffected relatives would carry the same candidate variants. The expected reduced penetrance implies that traditional segregation analyses will lack the power to identify true risk variants in the absence of a huge number of families. We therefore tested the putative causal variants in a population-based, case-control study followed by a confirmation analysis for the risk variants in a larger, independent nested case-control study and a meta-analysis combining data from the case-control studies.

## RESULTS

In this study, WES data were available for 160 affected men from 75 hereditary PCa (HPC) families (Table 1), which are a subset of families from the *PROGRESS* study [11]. We previously analyzed WES data from 19 of the 75 families [12]. In this current larger analysis, we combined WES data from our previous dataset of 19 families with an additional set of 56 families and then applied a new analysis strategy that focused specifically on identifying moderately penetrant causal variant candidates. The families are large with 70 having more than four PCa cases per family, 40 with more than six, and three with more than ten. One to six affected men per family had WES data with 31 families having WES data for multiple affected men. Twenty-two families had WES data for three or more affected men who are at least second-degree relatives of one another.

**Table 1:** Characteristics of the 75 high-risk families with WES data

Characteristic	No. of Families
Total number of affected men per family	
3 – 4	5
5 – 6	30
7 – 8	22
9 – 10	15
11 – 18	3
Mean age at diagnosis per family	
50 – 54.9	3
55 – 59.9	7
60 – 64.9	30
65 – 69.9	28
70 – 79.5	7
Number WES cases per family	
1	44
2	2
3	13
4	8
5	7
6	1
Max number WES cases who are ≥ 2^nd^ degree relatives	
0	45
2	8
3	17
4	5

**Figure 1:**
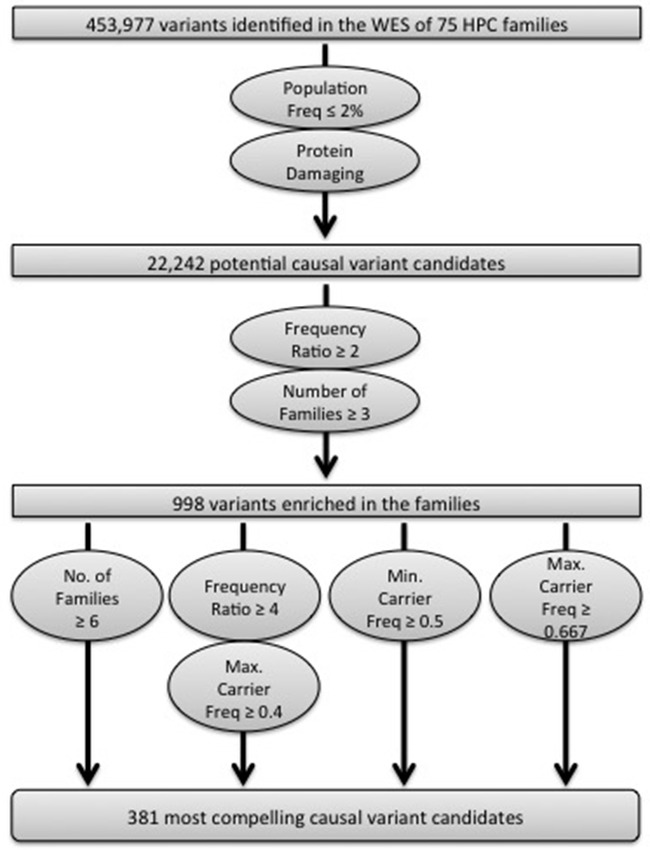
Schematic of the analysis strategy implemented to identify the most compelling variants for follow-up analyses

After calling sequence variants in all samples together and performing data quality filtering, 453,977 high quality variants were identified. Putative causal variants were selected for follow-up based on several criteria (Figure 1; see Supplemental Methods for details). Briefly, we selected variants with a population frequency ≤ 2% in all populations available at the time (n = 11), which included the NHLBI GO ESP (http://evs.gs.washington.edu/EVS/) and 1000 Genomes Project (http://www.1000genomes.org) published and exome datasets. Variant Effect Predictor was utilized to predict the protein impact of the variants in order to interrogate the consequence of each variant in all Ensembl transcripts (http://useast.ensembl.org/Tools/VEP). Both high impact variants (stop gained/loss, start loss, frameshift, and splice site alterations) and missense variants with a SIFT deleterious score [13] and/or PolyPhen2 probably/possibly damaging scores [14] were included. After the population frequency and protein impact filters, 22,242 variants remained.

The variants enriched in the WES dataset were then determined. One affected individual from each of the 72 families of European ancestry was selected, prioritizing men with aggressive and/or early-onset disease, and the observed frequency in these 72 men was compared to the NHLBI GO ESP European-American or 1000 Genomes European population frequencies (see Supplemental Methods for details). This allowed us to calculate what we termed the *frequency ratio*. Individual variants with a frequency ratio ≥ 2 that also segregated in at least three families were retained, resulting in 998 total variants (Figure 1).

One additional metric was utilized to select variants for follow-up: the average affected carrier frequency. Given the complex issues of genetic heterogeneity and incomplete penetrance, the average carrier frequency per variant was calculated, instead of the frequency in only the individuals with WES data. Determining the carrier frequency with all affected men maximized the informativeness of the 75 families, particularly the 44 families with only one WES individual. To calculate the average carrier frequency, we first genotyped 336 additional relatives from the 75 WES families using the 700K OmniExpress BeadChip (Illumina, San Diego, CA) for a total of 373 affected men. After integrating the WES and array-based SNP haplotype data (see Supplemental Methods for details), we determined how many affected men in each family could potentially carry the alternate allele. Depending on which WES individual(s) had the alternate allele, we were able to assign the alternate allele to either one or two possible haplotypes. Since some families could have two haplotypes that potentially carry the alternate allele, both a maximum and minimum possible carrier frequency per family was calculated, which would be the same value in situations where we were able to assign the alternate allele to a single haplotype within a family. These values were then averaged across all variant carrying families to generate either the maximum or minimum average carrier frequencies.

In order to identify the most compelling candidates for follow-up, we varied the three metrics, the frequency ratio, the number of families segregating the variant, and the average carrier frequency (Figure 1). All 105 variants that segregated in ≥ 6 families were chosen, irrespective of the average carrier frequency (Supplemental Table S2). We also retained variants with a frequency ratio ≥ 4, where the maximum average carrier frequency in families that carried the variant was predicted to be ≥ 40% (n = 215). Finally, we incorporated variants with the strongest apparent segregation among affected men within the families using two average carrier frequency thresholds. We chose variants with a minimum average carrier frequency ≥ 50% (n = 75), since the true average carrier frequency can only be higher than 50% for these variants. We also selected variants with a maximum average carrier frequency ≥ 67% (n = 97) because in a situation where all families have their highest possible carrier frequency, the true average carrier frequency would be the highest possible from the dataset. Some variants met multiple of the filtration criteria (n = 95). In total, 381 variants were selected for follow-up.

The 381 selected variants were genotyped in all men for whom DNA was available in the 75 WES families and in men of European ancestry from the two Fred Hutchinson Cancer Research Center (FHCRC) population-based, case-control studies of PCa [15, 16]. After quality and study design filters (see Methods for details), genotypes from 341 variants were available for 650 individuals from the 75 WES families (372 affected men, 238 unaffected men and 40 females) and 1,265 cases and 1,230 age-matched controls from the combined population-based studies.

Association between the alternate alleles of the 341 candidate variants and PCa risk in the FHCRC case-control study population was tested. Only variants that increased PCa risk were considered confirmed since each of the candidates was originally selected based on the carrier status of affected men within the high-risk families. Nine variants were associated with an increased PCa risk (nominal *P* < 0.05), including eight in genes not previously implicated in disease susceptibility (Table 2 and Supplemental Tables S3 & S4). Several variants had odds ratios (ORs) > 3.5, including *CHAD* p.Ala342Asp (OR = 3.51, 95% CI 1.30 – 9.49; *P* = 0.013), *BRD2* p.Ala605Pro (OR = 4.99, 95% CI 1.09 – 22.86; *P* = 0.038) and, as expected, *HOXB13* p.Gly84Glu (OR = 5.68, 95% CI 1.67 – 19.36; *P* = 0.0054).

**Table 2:** Association results for the nine variants with increased PCa risk^a^^,^^b^

		FHCRC				PLCO				Combined meta-analysis			
		1,265 cases and 1,230 controls				4,222 cases and 2,899 controls				5,487 cases and 4,129 controls			
Chr:Position	Variant	Ca/Co Freq^c^	OR^d^	95% CI^e^	P	Ca/Co Freq^c^	OR^d^	95% CI^e^	P	Ca/Co Freq^c^	OR^d^	95% CI^e^	P
1:22923859	*EPHA8* p.Pro607His	**2.50/0.94**	**2.70**	**1.20 – 6.07**	**0.017**	1.82/1.86	0.97	0.69 – 1.39	0.876	1.94/1.65	1.14	0.83 – 1.57	0.420
2:242695399	*D2HGDH* p.Ala225Thr	**2.46/1.06**	**2.37**	**1.23 – 4.56**	**0.010**	1.46/1.87	0.80	0.55 – 1.16	0.228	1.69/1.63	1.04	0.75 – 1.43	0.821
3:97868404	*OR5H14* p.Met59Val	**2.54/1.39**	**1.85**	**1.02 – 3.36**	**0.042**	2.42/1.93	1.27	0.92 – 1.78	0.157	**2.45/1.77**	**1.39**	**1.04 – 1.85**	**0.026**
6:32946119	*BRD2* p.Ala605Pro	**0.79/0.16**	**4.99**	**1.09 – 22.86**	**0.038**	0.62/0.73	0.87	0.49 – 1.57	0.640	0.66/0.56	1.09	0.63 – 1.86	0.765
17:46805705	*HOXB13* p.Gly84Glu	**1.42/0.25**	**5.68**	**1.67 – 19.36**	**0.005**	**1.11/0.31**	**3.78**	**1.94 – 8.28**	**0.0003**	**1.18/0.29**	**4.20**	**2.26 – 7.79**	**5.4×10^−6^**
17:48542714	*CHAD* p.Ala342Asp	**1.43/0.41**	**3.51**	**1.30 – 9.49**	**0.013**	1.23/0.97	1.28	0.81 – 2.06	0.292	**1.28/0.80**	**1.53**	**1.01 – 2.34**	**0.046**
19:11486354	*SWSAP1* p.Leu118Ile	**2.55/0.98**	**2.61**	**1.34 – 5.09**	**0.005**	1.44/1.31	1.12	0.75 – 1.70	0.593	1.70/1.21	1.41	0.99 – 2.00	0.055
22:20024596	*TANGO2* p.Ser17Ter	**2.77/1.47**	**1.93**	**1.08 – 3.42**	**0.026**	2.30/1.66	1.39	0.98 – 1.99	0.065	**2.41/1.60**	**1.52**	**1.13 – 2.05**	**0.006**
22:50873415	*PPP6R2* p.Arg103His	**2.07/1.06**	**2.02**	**1.03 – 3.96**	**0.040**	1.83/1.66	1.09	0.76 – 1.58	0.636	1.88/1.48	1.26	0.91 – 1.73	0.163

a Additional data are in Supplemental Table S4.

b Results with *P* < 0.05 are in bold.

c Case carrier frequency in percent/control carrier frequency in percent.

d Adjusted for age.

e 95% Confidence Interval.

The nine variants associated with an elevated PCa risk segregated in as few as three and as many as nine of the 75 WES families (Table 3), with six present in five or more families (*CHAD* p.Ala342Asp, *D2HGDH* p.Ala225Thr, *EPHA8* p.Pro607His, *HOXB13* p.Gly84Glu, *OR5H14* p.Met59Val and *SWSAP1* p.Leu118Ile). Almost half of the 75 families (n = 35) had at least one affected man who carried one of the nine risk alleles, and ten families had two or more variants carried by at least one affected man. Six of the nine variants (*BRD2* p.Ala605Pro, *CHAD* p.Ala342Asp, *EPHA8* p.Pro607His, *HOXB13* p.Gly84Glu, *PPP6R2* p.Arg103His and *TANGO2* p.Ser17Ter) had average carrier frequencies over 50% with a range from 50.4% – 64.3% (Table 3), which is similar to the 51% affected carrier frequency previously reported for *HOXB13* p.Gly84Glu [10].

**Table 3:** Results for the nine top-ranked variants after genotyping additional family members in the 75 high-risk families

		SIFT	PolyPhen2	Prostate Tissue	Final	No. Aff	No. Unaff	Avg Carrier
Chr:Position	Variant	Prediction - Score	Prediction - Score	Expression^a^	No. Fam	(n = 372)	(n = 238)	Frequency (%)^b^
1:22923859	*EPHA8* p.Pro607His	Deleterious - 0.02	Benign - 0.432	No	7	17	10	53.7
2:242695399	*D2HGDH* p.Ala225Thr	Deleterious - 0.04	Possibly - 0.774	Yes	9	24	11	40.1
3:97868404	*OR5H14* p.Met59Val	Deleterious - 0	Probably - 0.994	No	7	12	6	34.9
6:32946119	*BRD2* p.Ala605Pro	Tolerated - 0.37	Possibly - 0.870	Yes	3	8	1	56.7
17:46805705	*HOXB13* p.Gly84Glu	Deleterious - 0	Probably - 0.999	Yes	5	11	5	51.3
17:48542714	*CHAD* p.Ala342Asp	Deleterious - 0.02	Possibly - 0.608	Yes	5	13	1	50.4
19:11486354	*SWSAP1* p.Leu118Ile	Deleterious - 0	Probably - 0.991	Yes	5	11	3	46.0
22:20024596	*TANGO2* p.Ser17Ter	NA^c^	NA^c^	Yes	3	9	4	63.3
22:50873415	*PPP6R2* p.Arg103His	Tolerated - 0.17	Probably - 0.977	Yes	3	10	7	64.3

a Prostate tissue expression were determined using the GTEx Portal (http://www.gtexportal.org/).

b Supplemental Table S5 has the number of cases with the minor allele and the total number of cases for each segregating family.

c SIFT and PolyPhen2 do not make predictions for nonsense mutations.

The nine risk-associated variants were then genotyped and analyzed in 4,222 cases and 2,899 controls from an independent nested case-control study within the Prostate, Lung, Colorectal, and Ovarian (PLCO) Cancer Screening Trial [17, 18]. For overall associations (Table 2), *HOXB13* p.Gly84Glu was significant (OR = 3.78, 95% CI 1.94 – 8.28; *P* = 0.0003) and *TANGO2* p.Ser17Ter was borderline significant (OR = 1.39, 95% CI 0.98 – 1.99; *P* = 0.065). We then performed a meta-analysis of the FHCRC and PLCO study results (Table 2). Two of the nine variants were significantly associated with risk, *HOXB13* p.Gly84Glu (OR = 4.20, 95% CI 2.26 – 7.79; *P* = 0.0000054) and *TANGO2* p.Ser17Ter (OR = 1.52, 95% CI 1.13 – 2.05; *P* = 0.0062). Two additional variants were suggestively associated with risk, *OR5H14* p.Met59Val (OR = 1.39, 95% CI 1.04 – 1.85; *P* = 0.026) and *CHAD* p.Ala342Asp (OR = 1.53, 95% CI 1.01 – 2.34; *P* = 0.046), while the result for *SWSAP1* p.Leu118Ile (OR = 1.41, 95% CI 0.99 – 2.00; *P* = 0.055) was borderline. Based on the filtration strategy, two of the four associated variants, *HOXB13* p.Gly84Glu and *OR5H14* p.Met59Val, were initially chosen for further consideration because they had a frequency ratio ≥ 4 with a maximum average carrier frequency ≥ 40%. The *CHAD* p.Ala342Asp and *TANGO2* p.Ser17Ter variants were included because each had a minimum carrier frequency ≥ 50%. In the 75 high-risk families, three of the four risk associated variants were present in ≥ 5 total families (Table 3). *CHAD* p.Ala342Asp and *HOXB13* p.Gly84Glu segregated in five families each and *OR5H14* p.Met59Val in seven families. *TANGO2* p.Ser17Ter segregated in three families and had the highest average carrier frequency among the four associated variants at 63%.

A polygenic risk score using the four meta-analysis PCa risk associated variants was calculated (Table 4). The presence of any one of the four risk alleles was associated with a 1.63-fold increased risk of PCa (95% CI 1.36 – 1.95; *P* = 1.4 × 10^−7^). Co-occurrence of at least two of the four low-frequency risk alleles was observed in 17 cases and only three controls, and was associated with an even higher risk estimate (OR = 4.25, 95% CI 1.20 – 14.97; *P* = 0.024). Eight of the 17 cases with multiple variants carried the *HOXB13* p.Gly84Glu variant with seven of the eight carrying the combination of *HOXB13* p.Gly84Glu and *TANGO2* p.Ser17Ter. One family co-segregated both variants as well. This combination was not observed in any controls. When compared to cases and controls without either variant, the presence of both *HOXB13* p.Gly84Glu and *TANGO2* p.Ser17Ter occurred more often in cases (Fisher's exact *P* = 0.019). The observed number of cases with both *HOXB13* p.Gly84Glu and *TANGO2* p.Ser17Ter was also more than expected given the meta-analysis case frequencies for the variants (observed n = 7, expected n = 1.6, Fisher's exact *P* = 0.016).

**Table 4:** Genetic risk score for the four PC risk associated variants from the meta-analysisa

Number of Risk Alleles of Controls	Number of Cases	Percent of Cases	Number of Controls	Percent of Controls	OR^b^	95% CI^c^	P
0	5,087	92.98	3,928	95.60	1.00		Reference
1	367	6.71	178	4.33	1.58	1.31 – 1.91	1.0 × 10^−7^
2 or 3	17	0.31	3	0.07	4.25	1.20 – 14.97	0.024
≥1	384	7.02	181	4.40	1.63	1.36 – 1.95	1.4 × 10^−7^

a Only cases and controls with genotypes available for all four variants are included.

b Meta-analysis of age adjusted data.

c 95% Confidence Interval.

Subset analyses were then performed, stratifying by PCa first-degree family history in the FHCRC and PLCO studies separately and when combined in a meta-analysis (Table 5 and Supplemental Table S6). In the combined meta-analysis, the *TANGO2* variant was significantly associated with risk in men without family history (OR = 1.65, 95% CI 1.18 – 2.28; *P* = 0.0025). In the strata with a positive family history, although based on small numbers, we observed no difference between cases and controls (*P* = 0.93). This was the result of a higher frequency of the *TANGO2* variant in PCa family history positive controls (Table 5), which is not unexpected given our original hypothesis about unaffected relatives being likely carriers of moderately penetrant variants. *HOXB13* p.Gly84Glu was associated with disease in men without PCa family history (OR = 3.61, 95% CI 1.87 – 6.97; *P* = 0.00013) and in men with PCa family history (Fisher's Exact P = 0.032). *OR5H14* p.Met59Val was associated with risk in men without PCa family history (OR = 1.39, 95% CI 1.02 – 1.90; *P* = 0.036), but not in men with PCa family history (OR 1.47; *P* = 0.427). *CHAD* p.Ala342Asp was not associated with risk after stratifying by family history. Several of the subset analyses, including the tests with *CHAD* p.Ala342Asp, were likely underpowered given the sample size and the variant frequency.

**Table 5:** Meta-analysis association results after stratifying by first-degree family history of PCa^a^^,^^b^

Variant	Family	Cases (n = 5,392)			Controls (n = 4,056)			OR^d^	95% CI^e^	P
	History	Non-carrier	Carrier	Freq^c^	Non-carrier	Carrier	Freq^c^			
*HOXB13* p.Gly84Glu	No	4596	49	1.05	3697	11	0.30	**3.61**	**1.87 – 6.97**	**0.00013**
	Yes	733	13	1.74	340	1	0.29	na^f^	na^f^	0.032^g^
*TANGO2* p.Ser17Ter	No	4531	114	2.45	3655	56	1.51	**1.65**	**1.19 – 2.28**	**0.0025**
	Yes	728	17	2.28	333	8	2.35	0.96	0.41 – 2.28	0.928
*OR5H14* p.Met59Val	No	4526	113	2.44	3645	65	1.75	**1.39**	**1.02 – 1.90**	**0.036**
	Yes	724	20	2.69	335	6	1.76	1.47	0.57 – 3.82	0.427
*CHAD* p.Ala342Asp	No	4582	59	1.27	3675	31	0.84	1.48	0.95 – 2.30	0.080
	Yes	737	9	1.21	339	2	0.59	na^f^	na^f^	0.183^g^

a Supplemental Table S6 has the results for the FHCRC and PLCO studies separately.

b Results with *P* < 0.05 are in bold.

c Carrier frequency in percent.

d Meta-analysis of age adjusted data.

e 95% Confidence Interval.

f Meta-analysis was not be performed since one of the case-control studies had no control carriers (Supplemental Table S6).

g Fisher's Exact *P* value comparing case and control frequencies.

Finally, a meta-analysis stratifying by measures of aggressiveness where aggressive disease was defined as either Gleason score 8-10 or regional/distant stage disease was performed (Table 6, Supplemental Table S7). The *TANGO2* p.Ser17Ter variant was associated with aggressive disease in both the FHCRC (OR = 2.98, 95% CI 1.46 – 6.08, *P* = 0.0026) and PLCO (OR = 1.69, 95% CI 1.00 – 2.84; *P* = 0.048) case-control studies individually (Supplemental Table S7), and had a stronger risk estimate for aggressive disease (OR = 2.06, 95% CI 1.35 – 3.13; *P* = 0.00075) compared to non-aggressive disease (OR = 1.37, 95% CI 1.00 – 1.89; *P* = 0.047) in the combined meta-analysis (Table 6). *HOXB13* p.Gly84Glu was associated with both aggressive and non-aggressive disease in each study separately and in the meta-analysis (aggressive disease: OR = 5.86, 95% CI 2.49 – 13.77, *P* = 0.00005; non-aggressive disease: OR = 3.33, 95% CI 1.59 – 6.97; *P* = 0.0014). *OR5H14* p.Met59Val (OR = 1.43, 95% CI 1.06 – 1.93; *P* = 0.019) and *CHAD* p.Ala342Asp (OR = 1.54, 95% CI 1.00 – 2.38; *P* = 0.049) were associated with non-aggressive disease in the meta-analysis and while the lack of association for the aggressive disease comparison may be due to the small sample size, the risk estimates do not suggest a stronger association with aggressive disease for either the *OR5H14* or *CHAD* variants (OR = 1.23 and 1.65, respectively).

**Table 6:** Meta-analysis association results after stratifying by disease aggressiveness^a^^,^^b^

Variant	Disease Strata^c^	Non-carrier	Carrier	Freq^d^	OR^e^	95% CI^f^	P
*HOXB13* p.Gly84Glu	Control	4110	12	0.29	1.00		Reference
	Non-aggressive	4347	47	1.07	**3.80**	**2.02 – 7.17**	**3.62 × 10^−5^**
	Aggressive	1074	18	1.65	**5.54**	**2.71 – 11.32**	**2.68 × 10^−6^**
*TANGO2* p.Ser17Ter	Control	4059	66	1.60	1.00		Reference
	Non-aggressive	4298	97	2.21	**1.37**	**1.00 – 1.89**	**0.047**
	Aggressive	1055	35	3.21	**2.06**	**1.35 – 3.13**	**0.00075**
*OR5H14* p.Met59Val	Control	4051	73	1.77	1.00		Reference
	Non-aggressive	4278	111	2.53	**1.43**	**1.06 – 1.93**	**0.019**
	Aggressive	1066	23	2.11	1.23	0.77 – 1.99	0.387
*CHAD* p.Ala342Asp	Control	4087	33	0.80	1.00		Reference
	Non-aggressive	4336	56	1.28	**1.54**	**1.00 – 2.38**	**0.049**
	Aggressive	1076	14	1.28	1.65	0.89 – 3.08	0.112

a Supplemental Table S7 has the results for the FHCRC and PLCO studies separately.

b Results with *P* < 0.05 are in bold.

c Aggressive disease was defined as Gleason 8-10 or regional/distant stage.

d Carrier frequency in percent.

e Meta-analysis of age adjusted polytomous logistic regression data.

f 95% Confidence Interval.

## DISCUSSION

Similar to many common diseases, PCa is caused by a continuum from common, low-penetrance to rare, high-penetrance variants. Previous studies focused on the extremes of that continuum, identifying putative risk alleles from either high-risk families or from large case-control studies. Both approaches, however, entirely miss the important class of low-frequency variants of moderate risk. Using WES of high-risk families followed by validation in two independent case-control studies, we identify previously unknown alleles that increase disease risk in both high-risk families as well as men from the general population, regardless of family history. We believe this work has implications for understanding the genetic underpinning of other common, complex diseases.

In this study, we conducted an integrated analysis of WES data from 75 high-risk PCa families followed by evaluation of the most compelling causal variant candidates in the FHCRC case-control study population. Nine variants were found to be significantly associated with PCa risk (nominal *P* < 0.05), eight of which were in genes not previously implicated in PCa susceptibility. When the nine variants were analyzed in the independent PLCO nested case-control study, there was risk association evidence for the *HOXB13* p.Gly84Glu and *TANGO2* p.Ser17Ter variants. In a meta-analysis combining both the FHCRC and PLCO studies, four variants, *HOXB13* p.Gly84Glu, *TANGO2* p.Ser17Ter, *OR5H14* p.Met59Val, and *CHAD* p.Ala342Asp, were associated with increased PCa risk (*P* < 0.05). Inheriting two or more of the four risk variants was associated with a 4.25-fold increased PCa risk, which was largely driven by the co-occurrence of the *HOXB13* and *TANGO2* variants. We note however that the co-occurrence could be due to hidden population substructure in the cases and not the controls and that analysis of a larger collection of cases and controls is warranted. After stratifying by disease aggressiveness, *TANGO2* p.Ser17Ter was found to be associated with aggressive disease in the FHCRC and PLCO studies individually and displayed a stronger risk estimate for aggressive disease in the combined meta-analysis. When considered together, our results both replicate published findings and extend the list of moderately penetrant genes associated with risk of PCa, which, along with *HOXB13* p.Gly84Glu, are some of the first PCa variants to cross the bridge from family-based susceptibility to overall risk in the more general population.

For the three previously unidentified PCa risk genes, involvement in PCa susceptibility is plausible for *CHAD*, while for *TANGO2* and *OR5H14*, a model of causality is not yet clear. *CHAD* encodes a chondroadherin and a truncated version termed cyclicCHAD has been shown to inhibit breast cancer cell growth [19]. *OR5H14* is an olfactory receptor and while another olfactory receptor has been shown to promote PCa tumor development [20, 21], nothing is known about the function of *OR5H14*. The risk-associated variant in *TANGO2* is a stop-gain variant predicted to result in early truncation of multiple TANGO2 isoforms, including several expressed in the prostate (http://www.gtexportal.org/). While biallelic disruptions of *TANGO2* have been reported to cause pediatric metabolic myopathies [22, 23], the function of TANGO2 remains unknown and the metabolic phenotype is ascribed to the loss of the other TANGO2 isoforms that are not altered by the *TANGO2* p.Ser17Ter variant described here.

More than one hundred reported independent loci have been associated with PCa risk through either GWAS or linkage analyses of high-risk families [4]. Three of the four associated variants from our meta-analysis are within published PCa linkage peaks. *HOXB13* p.Gly84Glu and *CHAD* p.Ala342Asp are within the 17q21-22 linkage region [24–26]. *TANGO2* p.Ser17Ter is within a linkage peak at 22q11 that we previously identified in an analysis that incorporated disease aggressiveness in the *PROGRESS* families [27]. However, it is difficult to define the boundaries of linkage peaks, since many studies, including our own, were done with low resolution scans resulting in megabase-sized peaks. Thus, a more compelling strategy was to compare the four variants to previously replicated GWAS loci [5]. Only one of the four variants, *TANGO2* p.Ser17Ter, is within 500 kb of any of the 100 confirmed PCa GWAS loci and none are within 250 kb. Thus, this approach brings to the fore variants in genes/loci that have not been previously found in other datasets to be associated with increased PCa risk.

Our data suggest that the *CHAD* p.Ala342Asp variant could account for some portion of the unexplained linkage signal at 17q21-22 [28]. Only a portion of the 17q peak is accounted for by *HOXB13* p.Gly84Glu, and the rest is not explained by variants in either *BRCA1* [29] or *BRIP1* [30]. The *CHAD* and *HOXB13* variants segregate in different families and never co-occur in individuals from either the FHCRC or PLCO datasets. Recently, Johnson et al. used a candidate gene approach to analyze 11 high-risk families and found seven 17q21-22 variants that completely (n = 2) or partially (n = 5) co-segregated with disease in one family each [28]. To date, however, association with PCa risk for the six variants other than *HOXB13* p.Gly84Glu has not been evaluated. One of the five variants that partially co-segregated with disease in a single pedigree in the published dataset [28] was the *CHAD* p.Ala342Asp variant found in this analysis, providing additional evidence that this variant may contribute to the 17q21-22 linkage signal.

The filters used for selecting the candidate variants in this study were designed to highlight moderately penetrant variants that were enriched in the WES data from the 75 families. These criteria were certain to miss some variants, including those that are of either higher population frequency, segregating in less than three families, or simply not present in the dataset of 75 high-risk families, which is a relatively restricted dataset given the number of predicted PCa risk variants. In fact, if we select variants with population frequency less than 2%, that are high impact, and on the COSMIC cancer gene list (http://cancer.sanger.ac.uk/cosmic/curation), regardless of how many families segregate the same variant, there are 37 variants (Supplemental Table S8). Ten of these variants are in genes involved in DNA repair, including four *ATM* variants in one family each and two *BRCA2* variants segregating in three and one family respectively. For this study, we chose filters to allow for the optimal chance that the selected variants, while low-frequency, would be sufficiently common to be observed in the more general population. Applying the same stringent criteria to a distinct dataset, adding individuals/families to our existing dataset, or applying a different methodology, for example pVAAST [31], would likely highlight additional variants or genes with predicted involvement in PCa risk. These studies are necessary since the large number of failed linkage studies, coupled with the large number of confirmed loci from case-control association studies, suggests that the known catalog of PCa risk-associated genes/loci is incomplete. Overall, our data suggests that a combination of the variants described here and other as yet to be identified moderately penetrant risk variants are key for understanding the genomic underpinnings of PCa regardless of declared family history status.

## METHODS

### Subjects

#### Hereditary PCa families

Seventy-five families selected for WES were from the previously described *PROGRESS* study [11]. WES of 19 families was previously published using different bioinformatic methods and analysis strategies [12]. Seventy-two of the families are of European ancestry and three are of other ancestry. In the 44 families with only one affected man sequenced, disease aggressiveness (i.e., Gleason score 8-10 or regional/distant stage or death from PCa) was utilized to select the sequenced individual and then, when needed, early-onset PCa (≤ 65 years). In the 31 families with two or more affected men sequenced, selection of cases was designed to maximize the number of sequenced cases who are most distantly related, giving preference to cases with aggressive disease followed by early-onset disease.

#### FHCRC case-control study

Study participants were men of European ancestry enrolled in one of two population-based case-control studies of PCa risk factors carried out in residents of King County, Washington [15, 16]. There were 1,273 cases and 1,241 controls interviewed that had DNA available for genotyping.

#### PLCO case-control study

Study participants were prostate cancer cases and controls of European ancestry from the PLCO Cancer Screening Trial, which was a randomized trial of screening methods for the early detection of prostate, lung, colorectal, and ovarian cancers [17, 18]. Male participants randomized to the screening arm underwent prostate specific antigen (PSA) testing annually for six years and digital rectal examination annually for four years. DNA was available for genotyping from 4,234 cases and 2,907 controls.

Informed consent was obtained from all study participants in the HPC family-based *PROGRESS* study, in the FHCRC population-based case-control studies, and in the PLCO Cancer Screening Trial. The research projects were reviewed and approved by the Institutional Review Board at the Fred Hutchinson Cancer Research Center and the National Human Genome Research Institute. For the PLCO study, the study was approved by the Institutional Review Board at each center and the National Cancer Institute. Analysis of the WES data was also approved by the Mayo Clinic's Institutional Review Board.

### WES

Capture and sequencing was performed using Agilent SureSelect Human All Exon 50Mb (Agilent, Santa Clara, CA) for 80 individuals in 19 families at the Center for Inherited Disease Research (CIDR) and with the Agilent SureSelect Human All Exon v4+UTRs for 80 individuals in 56 families at the Mayo Clinic Medical Genome Facility. For all 75 families, paired-end sequencing was performed on the Illumina HiSeq2000 (Illumina, San Diego, CA). Bioinformatic analysis was performed for all 160 affected men at the National Human Genome Research Institute. NovoAlign (http://www.novocraft.com/) was used to align sequences to the human reference genome build hg19. Post-alignment optimization was done with Picard (http://broadinstitute.github.io/picard/) and GATK [32]. Variant calling was conducted on all samples in aggregate using GATK UnifiedGenotyper [33, 34]. For variant quality filtering, we ran GATK VQSR filtering tranche 99.0 and above. The 700K OmniExpress BeadChip genotypes were converted into VCF format using ChipMap developed by Peter Chines (NIH, Personal Communication). Genotype quality was set to 13 according to 99.9% concordance with the SNP array genotypes. The median read depth was 64.5 (range 19 – 177).

### OmniExpress beadchip haplotypes

The Illumina 700K OmniExpress BeadChip was used to genotype 508 individuals from the 75 families, including all 373 affected men with DNA available and 135 unaffected men or women, to aid in haplotype prediction and to rebuild haplotypes of affected men for whom DNA was unavailable. SNP arrays were run at three different geographic locations, thus genotypes were called using Illumina's GenomeStudio as three separate projects to prevent clustering problems. Clusters were visualized for SNPs with heterozygous excess ≤ -0.6 and ≥ 0.4 and GenTrain scores ≤ 0.5. SNPs were removed if the call rate was less than 100% and if the minor allele frequency was ≤ 0.1%. We used PLINK [35] to select the set of SNPs (n = 206,513) with r^2^ < 0.8 within 100 SNP windows with a five SNP step. Haplotypes for the autosomes were predicted using Merlin [36], where we allowed for three recombination events between informative markers and chose the most likely haplotype vector.

### Candidate variant genotyping

The Fluidigm Access Array microfluidic PCR technology was used for follow-up genotyping in 3,168 individuals according to the manufacturer's instructions (Fluidigm, South San Francisco, CA). Briefly, primer pair sequence and multiplexing were designed utilizing the Fluidigm D3 Assay Design website with 379 out of 381 passing primer design parameters. Custom Illumina adaptors and barcodes were ligated to the products, allowing 1,536 samples to be pooled together. Two pools of 1,536 samples and one pool of 96 were run on the Illumina HighSeq2000 and MiSeq respectively. Samples selected for follow-up genotyping included 1,273 cases and 1,241 controls from the FHCRC population-based case-control studies and all affected (n = 373) and unaffected (n = 239) men with DNA available in the 75 families as well as 40 females to rebuild haplotypes of affected men without DNA available.

The Fluidigm Access Array bioinformatic analysis was performed on all 3,168 samples together. CutAdapt (https://pypi.python.org/pypi/cutadapt) was used to remove primer sequences. Reads were aligned to the human reference genome build hg19 using BWA [37]. Post-alignment optimization was performed with GATK [32]. Variant calling was conducted with all samples together using GATK UnifiedGenotyper [33, 34]. Genotype quality was filtered at 60. The average read depth per sample was 799.6 (range 188.3 – 1324.9), excluding the seven samples that completely failed. The average read depth per amplicon was 850.8 (range 15.5 – 1454.6) excluding the seven amplicons that failed. Genotype concordance was 99.9% between the 66 SNVs in 478 individuals with both the OmniExpress 700K SNP Array and Fluidigm Access Array data and was 99.8% between the 692 SNVs in 171 individuals with WES and Fluidigm Access Array data. Twenty individuals failed or were excluded due to a call rate < 70%, which was set according to concordance data. Twenty-seven variants were removed for either having a call rate < 70% (n = 12), a frequency > 2% in controls (n = 9) or no longer being present in at least three families (n = 6). The remaining 350 candidate variants were visualized in multiple BAMs using IGV [38]. Variants (n = 9) failed visualization for multiple reasons including having evidence for amplification of multiple regions and poor sequence quality due to nearby sequence context. After quality filters, 341 variants in 3,145 individuals remained for further analysis, including 1,265 cases and 1,230 controls from the FHCRC study. Characteristics of the cases and controls with genotypes available are presented in Supplemental Table S1. Within the 75 WES families, genotypes were available for 372 affected and 238 unaffected men and 40 females.

### Replication genotyping

The MassARRAY iPLEX system was used to genotype the nine variants in the PLCO study according to the manufacturer's instructions (Agena Bioscience, San Diego, CA).

### Case-control association analyses

An underlying dominant genetic model was assumed when analyzing the association between genetic variants and PCa risk. Homozygote carriers of the most common allele were classified as the reference group. Unconditional logistic regression was used to calculate odds ratios (ORs) and 95% confidence intervals (CIs) for the overall risk and family history stratified analyses. Polytomous logistic regression was used for the stratification by disease aggressiveness analysis. For all case-control analyses, the models were adjusted for age. The case-control statistical analyses were conducted using the R programming language (http://cran.r-project.org/) including metafor for the meta-analysis.

## SUPPLEMENTAL METHODS AND TABLES


















