# Supplementary material for: Whole exome sequencing in 75 high-risk families with validation and replication in independent case-control studies identifies TANGO2, OR5H14, and CHAD as new prostate cancer susceptibility genes
Source: Oncotarget. 2016 Nov 26;8(1):1495–507. doi: 10.18632/oncotarget.13646 (PMC5341753; doi:10.18632/oncotarget.13646)
Supplement: Supplementary file 1 [file oncotarget-08-1495-s001.pdf]

Whole exome sequencing in 75 high-risk families with validation and replication in independent case-control studies identifies *TANGO2*, *OR5H14*, and *CHAD* as new prostate cancer susceptibility genes

SUPPLEMENTAL METHODS AND TABLES

Candidate variant selection

Variant call frequency

Half of our data (80 individuals from 56 families) were generated using a capture kit, which included additional exonic regions. Variants within the additional regions may meet the other candidate variant selection criteria even if only in the 80 individuals. Therefore, our variant call frequency was lower than expected, excluding only variants with < 70 out of 160 samples genotyped.

Population frequency

Variant frequency information was obtained from six publically available datasets including the NHLBI GO ESP European American and African American datasets (http://evs.gs.washington.edu/EVS/) and the 1000 Genome Phase 1 European, African, Asian and American datasets (www.1000genomes.org). We also called genotypes and calculated variant frequencies for all 453,977 variants in the five 1000 Genome Exome datasets (European, African, Asian, American and South Asian), which was particularly useful for variants without published population frequencies. We only considered variants with frequencies ≤ 2% in all eleven populations.

Protein impact

In order to check for protein impact in any Ensembl transcript, variants were annotated using VEP (McLaren et al., 2010). The transcript with the highest predicted impact was utilized for further consideration. We selected variants predicted to be protein damaging by both SnpEff (Cingolani et al., 2012) and VEP, which included high-impact variants (stop gain/loss, start loss, frameshift and splice site acceptor/donor) and missense variants predicted to be either SIFT (Kumar et al., 2009) deleterious or PolyPhen2 (Adzhubei et al., 2010) probably/possibly damaging.

Frequency ratio

In order to determine which variants are occurring more frequently in the families compared to the general population, we calculated a frequency ratio, which is the observed frequency in the families divided by the European population frequency. The observed frequency in the families was calculated by selecting one affected man per family with European ancestry (n = 72), prioritizing men with aggressive and/or early-onset disease. The European population frequency chosen was from NHLBI GO ESP. If unavailable, the published 1000 Genomes Phase 1 EUR frequency was used and then if both were unavailable the calculated 1000 Genomes EUR Exome dataset was utilized. Variants with twofold enrichment were selected for further consideration.

Average carrier frequency

For each family, we calculated the potential affected carrier frequency for each variant. To begin, we identified the WES affected men in each family that carried the alternate allele. We then used Merlin to track haplotype flow in pedigrees based on identical by descent patterns. Comparing the WES information to the Merlin predicted haplotypes at those genomic locations, we identified the haplotype(s) that contained the alternate allele and determined the expected carriers of the target variant per pedigree. Next, taking the total number of affected men that had the alternate allele haplotype(s), we calculated the carrier frequency of the alternate allele for each family. Depending on which WES individual(s) had the alternate allele, we were able to assign the alternate allele to either one or two possible haplotypes. Since some families might have two possible alternate allele haplotypes, we calculated both a maximum and minimum carrier frequency per family and averaged these values across all variant carrying families to generate the average carrier frequencies. There were six families too large to run Merlin with all family members. In those families, we calculated the carrier frequency from the trimmed pedigree with the greatest number of affected men.

Variant visualization

All candidate variants were visually inspected in multiple BAMs within the IGV software (Thorvaldsdottir et al., 2013). We utilized multiple criteria to determine the likelihood that a variant was real. Visualization criteria included evaluating the position of the variant within the read (i.e., not always at the end), whether the variant was found in reads in both directions, if the allele fraction was not one third or less in all samples with alternate allele, checking that the variant was not within a region of high depth, assessing sequence context (i.e., not in the

middle of microsatellites or repeats), determining that the mapping quality of alternate allele reads were > 70 and not different between reads with and without the alternate allele, and whether the alternate allele was just in one of the capture sets when both capture sets have coverage.

Supplementary Methods References

1. McLaren W, Pritchard B, Rios D, Chen Y, Flicek P and Cunningham F. Deriving the consequences of genomic variants with the Ensembl API and SNP Effect Predictor. Bioinformatics. 2010; 26:2069-2070.
2. Cingolani P, Platts A, Wang le L, Coon M, Nguyen T, Wang L, Land SJ, Lu X and Ruden DM. A program for annotating and predicting the effects of single nucleotide polymorphisms, SnpEff: SNPs in the genome of Drosophila melanogaster strain w1118; iso-2; iso-3. Fly. 2012; 6:80-92.
3. Kumar P, Henikoff S and Ng PC. Predicting the effects of coding non-synonymous variants on protein function using the SIFT algorithm. Nat Protoc. 2009; 4:1073-1081.
4. Adzhubei IA, Schmidt S, Peshkin L, Ramensky VE, Gerasimova A, Bork P, Kondrashov AS and Sunyaev SR. A method and server for predicting damaging missense mutations. Nature methods. 2010; 7:248-249.
5. Thorvaldsdottir H, Robinson JT and Mesirov JP. Integrative Genomics Viewer (IGV): high-performance genomics data visualization and exploration. Briefings in bioinformatics. 2013; 14:178-192.

Supplemental Table S1: Characteristics of the FHCRC and PLCO case-control study participants

See Supplementary File 1

Supplemental Table S2: Selection characteristics for the 105 variants chosen due to segregation in six or more families.

See Supplementary File 2

Supplemental Table S3: Association results for the 341 variants in the FHCRC study of 1,265 prostate cancer cases and 1,230 controls

See Supplementary File 3

Supplemental Table S4: Association results for the nine top-ranked variants in full detail

See Supplementary File 4

Supplemental Table S5: Carrier frequency and carrier count of the risk allele in all families segregating the top nine variants.

See Supplementary File 5

Supplemental Table S6: Association results when stratified by first-degree family history of PCa

See Supplementary File 6

Supplemental Table S7: Association results when stratified by disease aggressiveness

See Supplementary File 7

Supplemental Table S8: High-impact COSMIC variants with population frequency <2%

See Supplementary File 8
